# How teachers’ dominant interpersonal behavior shapes student emotions in mathematics classrooms: a multimodal voice analysis and critical incident study

**DOI:** 10.3389/fpsyg.2026.1829569

**Published:** 2026-05-29

**Authors:** Wei Lin, Xintong Lai, Hongbiao Yin, Sihan Wang

**Affiliations:** 1 Teacher Education Department, Guangdong University of Education, Guangzhou, China; 2 School of Education, South China Normal University, Guangzhou, China; 3 Department of Curriculum and Instruction, Faculty of Education, The Chinese University of Hong Kong, Sha Tin, Hong Kong SAR, China; 4 Institute of Education, University College London, London, United Kingdom

**Keywords:** China, critical incident technique, mathematics achievement emotions, mathematics teaching, multimodal voice analysis, teacher interpersonal behavior

## Abstract

**Introduction:**

Teacher’s interpersonal behavior plays an active role in guiding students to mathematical learning. This exploratory study aim to investigate the complex relationship between teachers’ dominant interpersonal behavior and students’ achievement emotions in Chinese secondary mathematics classrooms.

**Methods:**

Moving beyond traditional self-reports, this study introduces a novel methodological framework that integrates the Critical Incident Technique (CIT) with multi-modal voice analysis.

**Results:**

Through in-depth interviews and acoustic analysis of classroom interactions involving 16 secondary students from four distinct school types in China, the findings reveal that students hold ambivalent emotional responses toward teachers’ dominant interpersonal behavior.

**Discussion:**

When the instructional structure inherent in teachers’ dominant behavior was conveyed through a supportive vocal tone, characterized by moderate pitch, melodic variation, and fluent pacing, students perceived it as valuable pedagogical guidance, which they associated with enjoyment and pride. In contrast, when the interpersonal dominance was expressed through an authoritarian vocal tone, marked by high, sharp pitch, rushed speech, and vocal tension, students perceived it as restrictive social control, which was associated with self-reports of shame and hopelessness.

**Conclusion:**

Grounded in control-value theory, these findings suggest that students’ emotional reactions are not monolithic but are shaped by the acoustic delivery of dominance and their subsequent appraisals of control and value. This study extends control-value theory by identifying interpersonal style as a critical antecedent and validates the utility of multi-modal voice analysis as a powerful tool for capturing the nuanced socio-emotional fabric of classroom interactions, highlighting important implications for teachers’ use of dominant interpersonal strategies in mathematics teaching.

## 1 Introduction

Teachers’ dominant interpersonal behaviors are characterized by high-control interaction patterns and instructional methods employed during their interactions with students. This high-control approach to teaching is fundamentally grounded in the “Three Centers Theory,” conceived by the distinguished German educator Johann Friedrich Herbart, which positions teachers as facilitators in the learning process. This interpersonal behavior primarily emphasizes direct instruction, repetitive practice of fundamental skills, and constructive feedback (Baghoussi, 2021; Ghafar, 2023). This structure can be clarified by understanding teachers’ dominant interpersonal behavior from two complementary perspectives (Tian and Shen, 2023). One perspective refers to a social interaction pattern characterized by high dominance in the classroom, rooted in the model proposed by Wubbels and Levy (1991). The other perspective conceptualizes dominance as a teaching style that provides clear directives and emphasizes teacher-led control over learning activities (Reeve and Cheon, 2014).

In a dominant teacher interaction classroom, educators play an active role in guiding students to recognize and apply mathematical concepts for problem-solving (Sengupta-Irving and Enyedy, 2015). However, many Western scholars have criticized the teachers’ dominant interpersonal behaviors, arguing that it is fundamentally mechanical. They assert that teachers who utilize this method tend to view mathematics as a static discipline, where learning and mastery are primarily achieved through repetitive practice of exercises. This perspective can undermine students’ interest in mathematics (Bature and Campus, 2020). In response to these criticisms, numerous countries have been gradually transitioning from teachers’ dominant to students’ dominant interactions (Muganga and Ssenkusu, 2019). The overarching goals of contemporary mathematics curricula emphasize the importance of students’ dominant instructional strategies, reflecting a growing global demand for individuals who possess advanced mathematical reasoning skills capable of applying mathematical modeling to address real-world problems (Emanet and Kezer, 2021). Despite these shifts, the teachers’ dominant interactions continues to be prevalent in many developing countries (Muganga and Ssenkusu, 2019).

In terms of teacher interpersonal behaviors, current research reveals several significant gaps. First, while existing studies have described teachers’ dominant interpersonal behaviors, there has been insufficient in-depth examination of how these behaviors specifically influence students’ emotional responses during learning across various educational contexts. Additionally, the literature on teachers’ dominant behaviors indicates considerable regional differences in teaching practices and their effectiveness (Muganga and Ssenkusu, 2019), underscoring the necessity for research that takes into account cultural and geographical factors. Moreover, many studies suggest a direct, linear relationship between teaching behaviors and emotional outcomes (Lin et al., 2020). However, this perspective fails to capture the complexity of how a single teacher behavior can elicit a diverse range of emotions in students, such as anxiety, shame, or enjoyment. There is also a notable lack of research that integrates teacher interaction theory with established theories on student emotions, leaving a gap in our understanding of how these elements interact and influence one another. Although previous research has examined students’ mathematics achievement emotions across various contexts (e.g., Pekrun et al., 2010; Goetz et al., 2016), it has largely overlooked how teachers’ interpersonal behavior is enacted through vocal tone and how students interpret such vocal cues during the learning process. Conceptually, this study also faces a limitation concerning teacher-centered pedagogy, as prior studies often conflates two independent dimensions: instructional structure (pedagogical clarity and guidance) and interpersonal dominance (social control and authority) (Serin, 2018; Woods and Copur-Gencturk, 2024). In Confucian-heritage contexts like China, students may value instructional structure as effective support for learning, yet perceive interpersonal dominance as a threat to their social face and personal autonomy (Choi and Han, 2023; Wan et al., 2022). This issue remains underexplored in Chinese mathematics classrooms. Addressing these specific gaps is essential for developing a comprehensive understanding of how teachers’ dominant behaviors shape student experiences in diverse learning environments. This understanding can ultimately lead to more targeted and effective instructional strategies.

Traditional research on achievement emotions has relied heavily on self-reports, which are vulnerable to recall bias and social desirability. Recently, automated emotion recognition has advanced, especially methods that infer emotions from speech and text (e.g., Jmour et al., 2021; Hossain and Muhammad, 2019), and affect-aware technologies show promise across domains. Yet the moment-to-moment link between teacher interpersonal behavior and student emotion remains underexplored, largely due to methodological limits. Teachers’ dominant interpersonal behavior is conveyed not only through words but also through paralanguage—tone, pitch, pace, and vocal energy (D’Mello and Gruber, 2021). The same question can sound encouraging or intimidating, prompting very different emotions. Likewise, a student’s voice can signal confidence or anxiety before they say so explicitly. Therefore, a more granular, objective, and dynamic approach is needed to understand how teachers’ dominant behaviors shape the emotional climate of mathematics classrooms. Based on Control-Value Theory (Pekrun, 2006) and prior research on interpersonal teacher behavior (Wubbels et al., 2014), this study proposes two hypotheses to address the above research questions:

*H1*: A supportive vocal tone (characterized by moderate pitch and fluent pacing) is expected to enhance students’ perceived control and positive value, which, in turn, amplifies their enjoyment and pride through these improved appraisals.

*H2*: An authoritarian vocal tone (characterized by a sharp pitch and rushed speech) is expected to undermine students’ perceived control and positive value, thereby triggering shame and hopelessness due to diminished appraisals.

To test these hypotheses, the present study addresses the following research questions:

(a) What emotional responses do students exhibit towards teachers’ dominant interpersonal behaviors in mathematics?

(b) How do teachers’ dominant interpersonal behaviors influence students’ achievement emotions during mathematics learning?

To address these research questions, the present study introduces a novel methodological framework, by which multimodal voice analysis was adopted to support the Critical Incident Technique (CIT) to capture students’ emotional responses. This approach aims to objectively and continuously measure the emotional undertones of teacher–student interactions in mathematics classrooms characterized by teachers’ dominant interpersonal behavior, thereby offering a more fine-grained and contextually grounded understanding of students’ achievement emotions.

## 2 Theoretical perspectives

To comprehensively understand how teacher behavior influences student affect, this study integrates two theoretical frameworks: the Model of Interpersonal Teacher Behavior and the Control-Value Theory of achievement emotions. The former provides a lens for classifying teachers’ dominant interpersonal behavior, while the latter offers a mechanism for explaining the divergent emotional outcomes that such dominance can provoke.

### 2.1 Teacher interpersonal behavior in mathematics classes in China

**Figure 1:**
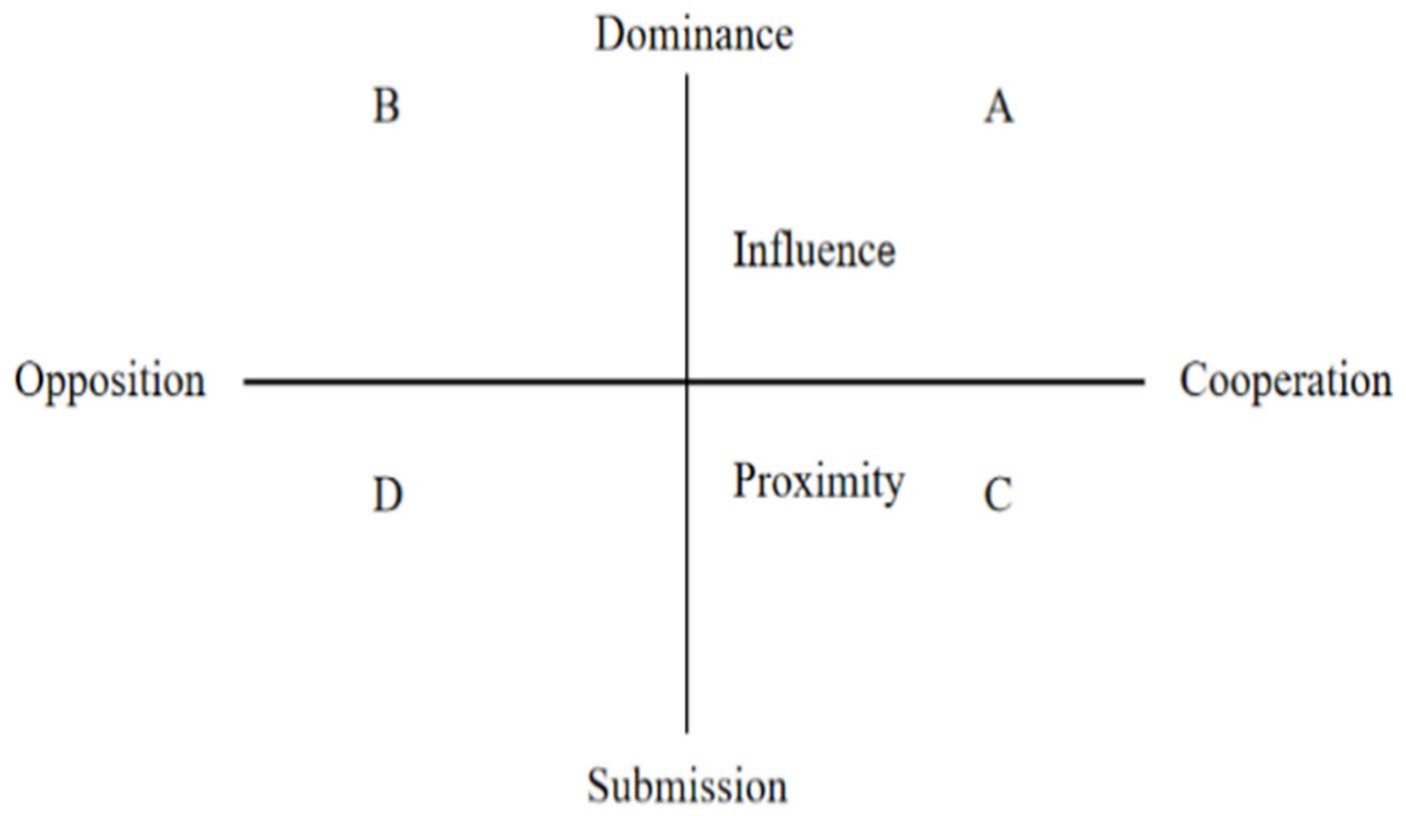
Leary’s model of interpersonal behavior.

The concept of teacher interpersonal behavior was first introduced by Wubbels et al. (2014) at the University of Utrecht in Denmark. Teacher interpersonal behavior refers to the interactions between teachers and students within the classroom setting. It is known as the Teacher Interpersonal Circle (IPC-T), and characterizes teachers’ behavioral tendencies by drawing on students’ perceptions of teachers’ interpersonal behaviors as enacted in the classroom. Influenced by early clinical medicine’s analysis of human interactions through dialogues and group discussions, researchers typically categorize these interactions into two main dimensions: influence and proximity. Within this framework, T. Leary developed a model that utilizes the “influence” dimension to assess who leads or controls the communication process in the classroom and to what extent. Specifically, “influence” is evaluated based on two factors: dominance (D) and submission (S). In parallel, Leary also examines the “proximity” dimension to gauge the level of cooperation or closeness between teachers and students during these interactions. This dimension is measured through cooperation (C) and opposition (O), collectively referred to as the “love-hate axis.” This framework aids in discussing the relationships between teachers and students, allowing researchers to analyze classroom interactions through a systems communication perspective (see Figure 1). This structure indicates that teachers who display dominant interpersonal behavior score highly on the “dominance” dimension, which refers to specific, observable actions. These include monopolizing student speaking time, minimizing question-and-answer exchanges, controlling the direction of topics, and labeling student responses that do not match a preset standard as “incorrect” (Husain and Mariana, 2026). Structurally, such teachers emphasize leadership and control by providing clear directives on task completion and learning goals, thereby shaping a classroom environment that prioritizes responsibility and compliance (Reeve and Cheon, 2014; Tian and Shen, 2023).

In mathematics education, and in line with Freire’s (2018) Pedagogy of the Oppressed, teacher–student interactions can also be differentiated by the extent of student involvement (Muganga and Ssenkusu, 2019). A substantial body of prior research on teacher–student interaction has focused on its effects on students’ mathematics achievement and learning motivation (Iotti et al., 2020). Other studies have examined how the quality of teacher–student relationships relates to students’ cognitive and non-cognitive attributes—such as general cognitive ability, self-esteem, and mathematics anxiety—and, in turn, how these factors influence lower-secondary students’ mathematics performance (Semeraro et al., 2020). While existing evidence indicates that teacher–student interaction bears on both mathematics attainment and affect, recent work has begun to question and explore how preadolescent and adolescent students perceive and experience teachers’ interpersonal behaviors. Scholars have underscored the need for longitudinal research to test age-related differences in the effects of teacher–student interaction (Boukari et al., 2022). In terms of cross-sectional scope, attention has turned to students of the same region and age but attending different types of schools and exhibiting different levels of prior attainment, probing differences in their perceptions of teacher interaction (Sun et al., 2018). At the same time, the influence of cultural variation on teachers’ interactive behaviors—and students’ culturally situated expectations of the teacher’s role—has remained a persistent focus (Zhang and Oetzel, 2006).

In China, traditional lecturing and rote memorization have increasingly given way to lower-control pedagogies such as project-based inquiry and small-group work (Dello-Iacovo, 2009). Nevertheless, compared with whole-class lecturing, these approaches are often more time-consuming and less predictable (Wang, 2011). As a result, several obstacles impede the shift toward student-centered teaching. First, top-down reforms tend to lose momentum as they diffuse through multiple layers of stakeholders (Zhang and Adamson, 2007). Second, strong parental pressure for examination success persists. Third, efforts to promote learner autonomy often clash with teachers’ preferred strategies for preparing students for high-stakes exams (Halstead and Zhu, 2009). In this context, student-centered approaches face substantial implementation challenges.

Given that Chinese classrooms typically operate within a fixed curriculum and timetable, high-control lesson structures can help ensure that required tasks are completed efficiently (Wang, 2011). At the same time, although high school students often achieve strong results in mathematics, many struggle with low motivation and confidence, drawing attention to the importance of students’ mathematical emotions (Lin and Yin, 2024). Historically, China’s centrally governed system has favored direct, teachers’ dominant interpersonal behavior, whereas many Western systems position teachers as facilitators—leading to the expectation that Western students would report greater enjoyment and interest. Experimental evidence suggests cultural moderation: Chinese and U.S. students of similar age exhibit different affective responses to the same controlling instruction (Zhou et al., 2012). These findings align with research on teachers’ interpersonal styles. High agency combined with high communion—the “warm demander”—is associated with greater enjoyment, whereas high agency paired with low communion—the “cold commander”—is linked to negative emotions such as anxiety (Pennings et al., 2018). Thus, students do not necessarily reject instructional control, and teachers’ dominant interpersonal behavior is not inherently detrimental to affect; its impact depends on relational cues, instructional clarity, and cultural meaning. To be specific, students tend to view instructional structure (clear guidance, logical sequencing) as a valuable tool for academic success, while reacting negatively to interpersonal dominance (authoritarian control, public criticism) that threatens their face and autonomy (Lin et al., 2025; Wan et al., 2022). Future research should disentangle agency from communion, consider cultural interpretations, and attend to developmental stage when assessing how teacher-led practices shape learners’ emotional experiences in mathematics.

### 2.2 Mathematics achievement emotions and control-value theory

Achievement emotions are defined as emotions associated directly with one’s achievement activities or achievement outcomes (Pekrun, 2006; Goetz et al., 2016). In order to elaborate achievement emotions, Pekrun (2006) employed control-value theory to analyze achievement emotions. “Control” means individuals’ intervention on mediators which affect the related results they perceive, in order to change their own behaviors and feelings. “Value” means the importance of activities and outcomes for the individual. According to control-value theory, as proposed by Pekrun (2006), a taxonomy has been introduced to categorize students’ achievement emotions across three dimensions. These dimensions distinguish emotional functions by assessing variations in control and value perceptions. First, prospective outcome emotions refer to emotions controlled by individuals to achieve success or avoid failure and focus on the possible impacts of the outcome on individuals. Prospective outcome emotions include positive emotions (e.g., anticipatory enjoyment—high control, hope—middle control, relief—low control) and negative emotions (e.g., anger—high control, anxiety—middle control, hopeless—low control). Second, retrospective outcome emotions concern the outcomes produced by individual’s control that depends on self, others, or external circumstances. Retrospective outcome emotions also include positive emotions (e.g., enjoyment—no related to control, pride—self feeling) and negative emotions (e.g., anger—no related to control, shame—self feeling) according to individuals’ value judgement. Third, activity-related emotions refer to emotions associated with individuals’ academic achievement and learning processes. Activity-related emotions include two high controlled emotions (e.g., enjoyment—positive value, anger—negative value) and a low controlled emotion, that is, frustration. Individuals focus on their own behavior instead of outcome when experiencing this type of emotions.

Researchers classified different kinds of mathematics achievement emotions with various levels of control and value appraisals. Pekrun (2006) took both object focus and valence focus into account and rendered a 2*2 taxonomy, grouping these emotions as follows (Goetz et al., 2016): (a) activity/positive (e.g., enjoyment of learning), (b) activity/negative (e.g., boredom experienced during learning, and anger about assignments or tasks), (c) outcome/positive (e.g., hope, pride), and (d) outcome/negative (e.g., anxiety, hopeless, shame). Pekrun (2006) also presented that two types of achievement emotions should be included in the outcome emotions. One is prospective anticipatory emotions (e.g., hope for success, anxiety of failure), and the other is retrospective emotions (e.g., pride or shame experienced after feedback of achievement). Students’ achievement emotions have proven to be affected by classes’ aggregate environment perceptions and different learning situations (Peixoto et al., 2017). Especially, Goetz et al. (2016) has found that students’ emotions would be influenced by their interactions with their teachers. For example, teachers’ demand, enthusiasm and passionate teaching could improve students’ academic performance by reducing their negative feelings such as boredom and anger. If teachers brought too much pressure to students, students would feel anxious and angry. From the student’s perspective, teachers’ vocal responses make their control-value appraisals observable. A pride-related vocal profile (e.g., strong, fluent speech) signals high perceived control and positive value, whereas a shame-related profile (e.g., weak, disfluent speech) signals low perceived control and negative self-value (Scherer, 2013).

Recent studies have examined how variables such as task difficulty (Decarli et al., 2025), teacher stress (Schonert-Reichl, 2017), teachers’ technological competence (Saccardo et al., 2024), and the phases of mathematical problem-solving (Cuisinier et al., 2010) influence students’ emotional experiences in the classroom. However, comparatively little attention has been paid to the role of teacher–student interactions, especially in high-control instructional settings in shaping students’ emotions. Existing evidence suggests that teacher-centered leadership behaviors can bolster positive achievement emotions (Lin et al., 2020). Yet it remains unclear whether the same behaviors elicit different emotional responses depending on contextual factors within the classroom. The empirical picture is mixed: some studies report no cross-cultural differences in positive emotions (Zhang and Oetzel, 2006), whereas others find that Chinese students report both higher enjoyment and higher anxiety in mathematics (Jia and Cheng, 2024). This uncertainty highlights the need to examine more closely how students’ emotional experiences are influenced by dominant teacher interactions in mathematics lessons.

### 2.3 Theoretical integration, cultural context, and operationalized hypotheses

#### 2.3.1 An integrated theoretical model

**Figure 2:**
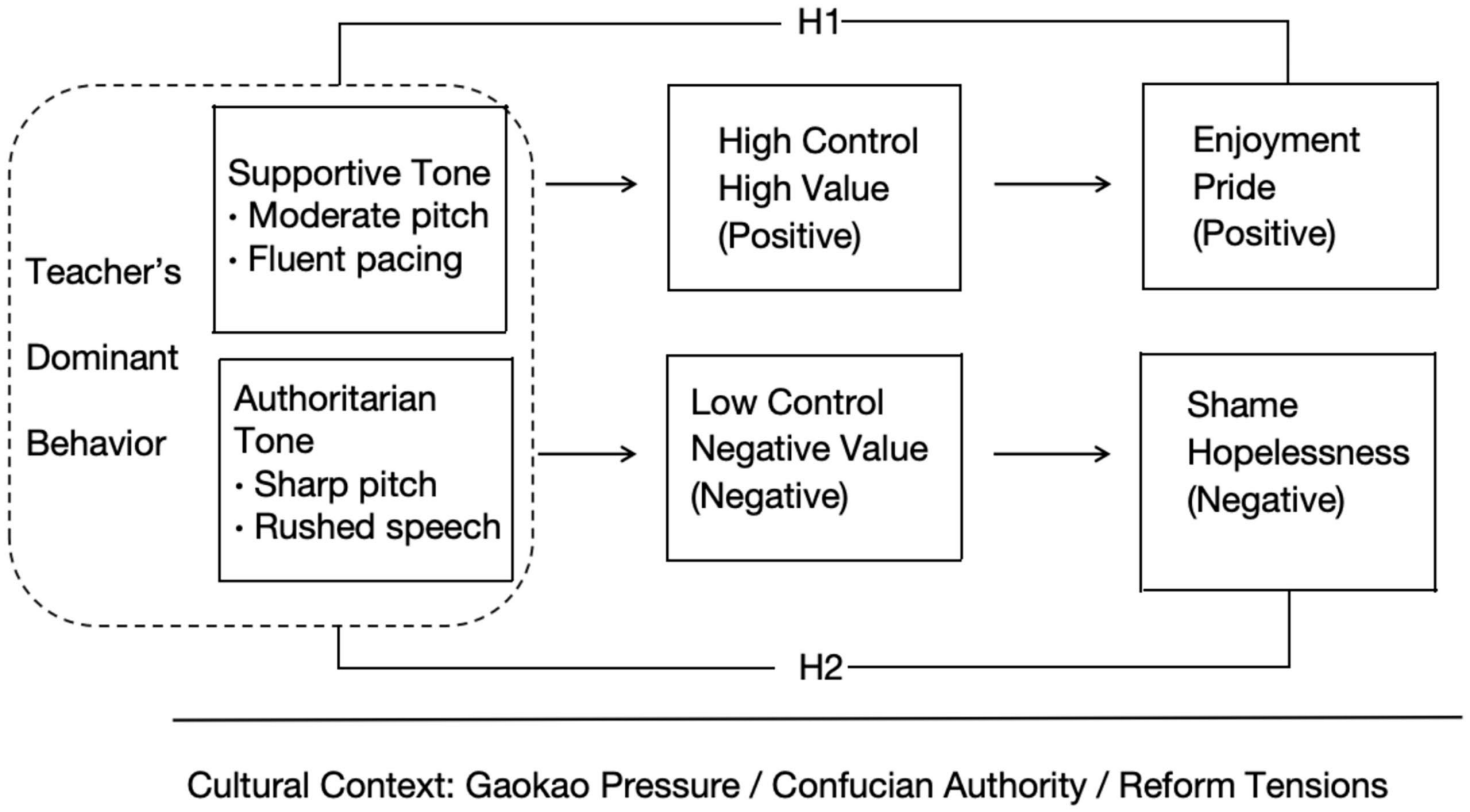
Integrated theoretical model: Pathways from teacher vocal tone to student achievement emotions via control-value appraisals.

Based on the complementarity of IPC-T and CVT, this model presents an integrated framework, proposing that teachers’ dominant interpersonal behaviors, particularly their acoustic delivery, function as situational antecedents that shape students’ appraisals of control and value, thereby eliciting specific achievement emotions. Figure 2 illustrates this integrated framework.

As illustrated in the model, the interpersonal dimension of dominance (cooperative versus oppositional) is operationalized through specific vocal parameters. These acoustic features are hypothesized to influence students’ control and value appraisals, which subsequently determine the quality and valence of their achievement emotions in mathematics classrooms. The model posits universal pathways from vocal features to emotions via appraisals; however, the interpretation of teacher dominance, and thus the resulting emotional outcomes, is likely moderated by cultural context.

#### 2.3.2 Cultural context: the Chinese learner paradox

The interpretation of teachers’ dominant interpersonal behavior in Chinese mathematics classrooms cannot be divorced from its cultural embeddedness. Research has long noted an emotional paradox in Chinese mathematics classrooms (Rao and Chan, 2009): teacher-dominated instruction, while yielding strong academic outcomes in international assessments (Watkins and Biggs, 1996), may also incur emotional costs in terms of constrained autonomy and reduced self-efficacy (Esakpaide, 2026). Students tend to accept teachers’ authoritative directives as a pathway to academic success (Goldschmidt et al., 2016), yet this acceptance may come at an emotional cost. In societies with high power distance, student-teacher communication tends to be implicit and indirect, and students are inclined to refrain from publicly questioning or challenging teachers’ authority (Loh and Teo, 2017). Two intersecting cultural forces shape this ambivalent response.

First, the “Gaokao” imperative. The high-stakes Zhongkao and Gaokao examinations in China sustain teacher-centered instruction as an efficient means of content coverage and exam preparation, an approach shown to positively influence motivation and achievement (Lavy and Schlosser, 2011; Schwerdt and Wuppermann, 2011). However, this exam-oriented system simultaneously constrains teachers’ capacity to foster student autonomy (Cheng and Ding, 2021). Consequently, teachers’ dominant behaviors are often perceived as pragmatically necessary for academic success, creating a context in which control is accepted despite potential emotional costs.

Second, the tension between Confucian tradition and student-centered reforms. Growing up in a collectivist society, Chinese adolescents are deeply shaped by Confucian traditions emphasizing respect, interpersonal harmony, and deference to teachers (Qin et al., 2025). The teacher–student relationship is traditionally characterized by high power distance, in which teacher authority is associated with respect, knowledge, and moral virtue (Alqarni, 2022). However, contemporary curriculum reforms, often influenced by Western educational philosophies, advocate student-centered pedagogies that emphasize open-ended questioning and supportive feedback. Yet, implementation of these reforms faces challenges, as established classroom norms continue to shape relatively quiet or less participatory student engagement (Hao et al., 2026), creating tensions between traditional expectations and modern pedagogical ideals. This cultural ambivalence helps explain why the same dominant teacher behavior can elicit contradictory responses: students may simultaneously value teacher control as effective guidance (rooted in Confucian respect) and resent it as restrictive (driven by emerging autonomy-oriented expectations).

With this cultural situatedness in mind, the specific hypotheses linking acoustic features to emotional outcomes within the Chinese classroom context can be further operationalized.

#### 2.3.3 Operationalized hypotheses

Based on the integrated theoretical model and the cultural considerations outlined above, the following hypotheses are operationalized with reference to the acoustic features identified in Table 1 and the emotional categories presented in Table 2:

**Table 1:** Reference acoustic ranges for six emotions from CASIA database.

Emotion	Mean pitch (Hz, p05-p95)	Mean loudness (p05-p95)	Spectral flux (p05-p95)
Happy/enjoyment	132–341	0.326–0.935	0.118–0.646
Surprise	150–361	0.301–1.077	0.128–0.928
Angry	144–332	0.420–1.178	0.203–1.044
Fear/shame	114–312	0.315–0.806	0.122–0.493
Sad/hopeless	105–259	0.205–0.664	0.066–0.400
Neutral	100–274	0.389–0.785	0.161–0.461

p05–p95 = 5th to 95th percentile range across all utterances per emotion.

**Table 2:** Results of relational coding and multimodal voice data.

Primary emotional categories	Subcategories	Category connotations	Key vocal correlates in student speech
Enjoyment	Passionate learning	Students experience enthusiasm for learning in mathematics.	Spontaneous laughter; responsive vocal backchannels (e.g., “mm-hmm”); relaxed and rhythmic breathing sounds.
	Understanding well	Students are able to understand the explanations provided by teachers.	
	Effective management	Students perceive that teachers’ management fosters a conducive learning environment.	
Pride	Emphasis on key concepts	Students believe that teacher effectively addresses the key points and challenges during the lesson.	High, stable energy; fluent, confident speech with few disfluencies; slower, more deliberate pacing (as if savoring the moment).
	Alleviation of difficulties	Instructional practices are viewed as beneficial in aiding students to resolve their misunderstandings.	
	Mastery of mathematics	Students feel that mathematics examinations or assignments are easy to handle, resulting in their satisfactory performance.	
	Serving as a peer tutor	Students are able to teach their peers.	
Shame	Fear of embarrassment	Students’ worry regarding to potential blame for their unsatisfactory mathematics performance.	Low energy; slow speech rate; high pause-to-speech ratio; elevated jitter (voice tremor).
	Feelings of shame and discomfort	After receiving poor grades or criticism from teachers	
	Embarrassment and shame	Following ridicule from classmates or teachers	
Anger	Being irritated	Students face insults and attacks from parents, teachers, and others in mathematics learning.	Sudden spikes in energy and pitch; harsh voice quality (abnormal spectral features).
	Emergence of dissatisfaction	Students doubts and dissatisfied regarding the professionalism of teachers.	
Hopeless	Lack of autonomy	Students believed that mathematics learning was an unattainable task for them	Continuously low energy; very low pitch variability (monotone); slow, drawn-out syllables.
	Hopelessness in mathematics learning	Students endure repeated setbacks in their mathematics learning.	

*H1* (Supportive Tone → Positive Emotions): We expect that a cooperative (supportive) vocal tone, operationalized as moderate pitch, melodic variation, and fluent pacing (Table 1), will be associated with students’ enjoyment and pride (Table 2). This tone highlights instructional structure with clear and well-paced guidance, which enhances students’ value perceptions of mathematics learning.

*H2* (Authoritarian Tone → Negative Emotions). We expect that an oppositional (authoritarian) vocal tone, operationalized as high/sharp pitch, rushed speech, and vocal tension (Table 1), will be associated with students’ shame and hopelessness (Table 2). By comparison, another tone conveys interpersonal dominance through hurried delivery and coercive control, impairing students’ sense of control and damaging their social face.

These hypotheses are tested using the multimodal methodological framework described in Section 3, with triangulation between CIT-derived emotional categories (Table 2) and acoustic parameters (Table 1).

## 3 Methods

### 3.1 Participants in study

This study is supported and approved by the Research Ethics Review Committee of National Ministry of Education in China. All participants, along with their parents/guardians, provided written informed consent. Participants were informed of their right to withdraw at any time, and all data were anonymized to ensure confidentiality. Participants were selected according to the following explicit inclusion criteria: (a) currently enrolled in Grades 7–9 in Shenzhen, Guangdong Province; (b) exposed to mathematics instruction delivered by teachers exhibiting dominant interpersonal behaviors for a minimum of six consecutive months prior to participation; and (c) having no diagnosed learning disabilities or emotional disorders that could interfere with the recall or articulation of emotional experiences.

The method of an embedded case study was used in this study. By using purposive sampling strategy, four different school cases were selected to participate in this study, which were private school, public school, prestigious school, and nine-year consistency school in Shenzhen, Guangdong province. They were selected due to their representation of the predominant school typologies in China, and the inclusion of mathematics instructors boasting notable expertise, including members affiliated with the Master Teacher Studio, leaders within the field of mathematics education, and novice educators. In total, 16 students from 4 school cases were involved in this study. This sample size aligns with Critical Incident Technique guidelines, which achieve thematic saturation with 10–20 participants (Flanagan, 1954). The 16 participating students were in grades 7–9 (ages 13–15), an early adolescent period critical for the development of academic identity and emotional regulation. Table 3 shows the background information of the participants.

**Table 3:** Background information of participants and interview schedule in the study.

Background information of the participants				Name (pseudonym)	Gender	Years of teaching	School type
David	M	12	Nine-year school
Ben	M	6	
Jack	M	25	
Alice	F	12	
Ricky	M	10	Public school
Crystal	F	10	
Jane	F	10	
Tony	M	28	
Angela	F	25	Private school
Cindy	F	25	
Hugo	M	13	
Daisy	F	20	
Emily	F	15	Key school
Gloria	F	15	
Susan	F	5	
Michel	F	5	

Interview schedule
Explore views about teachers’ dominant interpersonal behavior in relation to ‘emotional patterns’ For example: When did you feel happy or anxious in your math classes? Can you tell me a story about a wonderful math class you have experienced?
Explore views about teachers’ dominant interpersonal behavior in relation to ‘emotional responds’ For example: What did you feel about your math teachers’ teaching or interaction?
Explore views about teachers’ dominant interpersonal behavior in relation to ‘emotional adjustment’ For example: What can your math teacher do to make you love math more? Can you give some suggestions to your math teachers about their teaching and interaction?

### 3.2 Data collection and analysis

**Figure 3:**
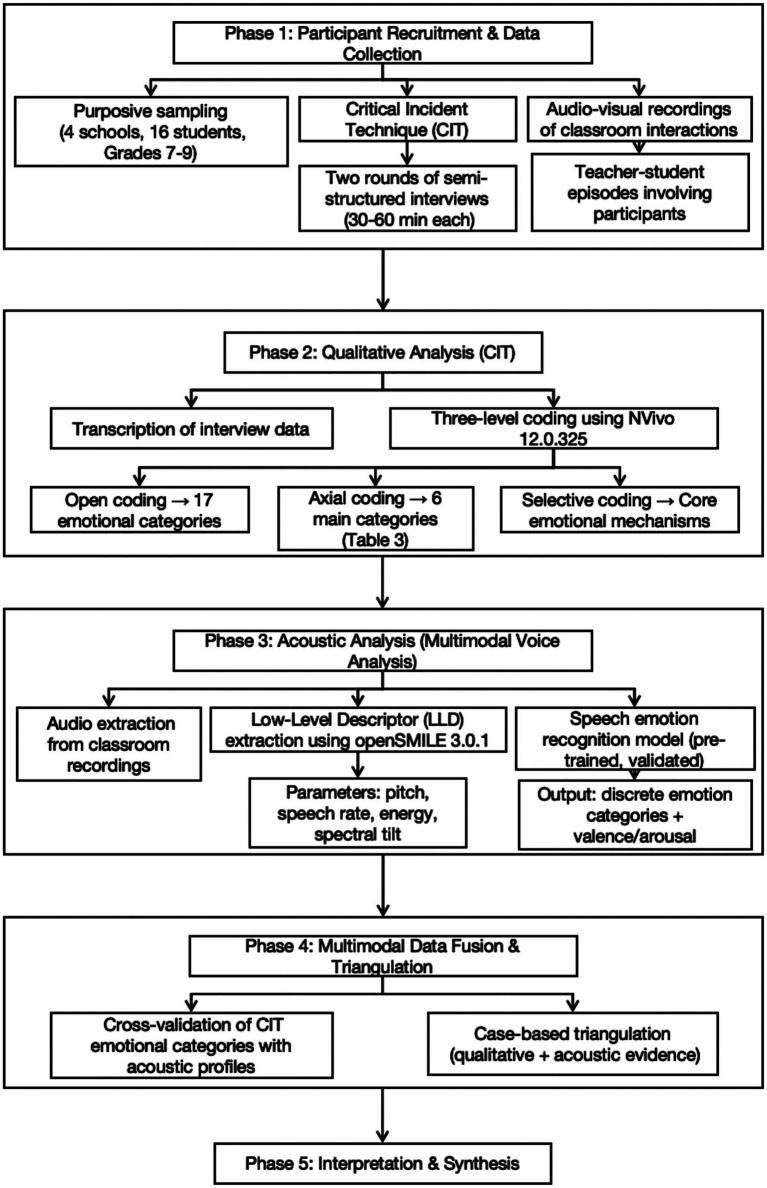
Schematic overview of the sequential methodological procedures.

Figure 3 presents a schematic overview of the sequential methodological procedures employed in this study, from participant recruitment through to multimodal data integration. Firstly, this study employs the critical incident technique (CIT), a seminal qualitative research method, for data collection and analysis (Flanagan, 1954). The classification of critical incidents is based on the emotional significance and meaning that these events impart to the participants, highlighting the importance of understanding students’ emotional experiences in their learning processes. Two rounds of semi-structural interviews with different foci were conducted in Chinese. The interviews were conducted using a scheduled list of open-ended questions designed to encourage participants to share their personal stories and feelings regarding the high-control teacher interaction behaviors method. The interview schedule aimed to explore participants’ perspectives on this method in relation to their emotional patterns, emotional responses, and emotional adjustments (see Table 3). All interviews were carried out face-to-face in a location (mainly the schools or cafés near school) that was convenient and comfortable for the participants, and each interview lasted 30 to 60 min. With the permission of the participants, each interview was audio-recorded and further transcribed. Secondly, we implemented a multimodal voice data collection procedure to support methodological triangulation. It allows for the analysis of moment-to-moment emotional responses that may not be fully captured through retrospective interviews. Using purposive sampling, we selected audio-visual recordings of key teacher–student interactions involving the participants and subjected them to multimodal voice analysis. This component examines how vocal features of teacher talk—such as encouraging, neutral, or interrogative tone—in teacher-dominated mathematics classrooms shape the immediate emotions detectable in students’ vocal responses.

#### 3.2.1 CIT data analysis: three-level coding process

CIT Data were analyzed using NVivo 12.0.325 software, and a three-level coding procedure was employed for data processing. Through systematic coding in phases 1 to 3, the analysis showed no new concepts or changes in relationships between existing ones. This confirms the study’s interpretive validity and strengthens the reliability of its findings. Phase 1 involved open coding, which focused on the conceptualization of raw data. This research employed a line-by-line reading of the data, along with concept naming and coding integration, to identify events that elicited emotional responses from students during mathematics learning. Subsequently, initial concepts were distilled, including aspects such as teacher praise, the simplicity of mathematical problems, teachers’ enthusiasm during lessons, and the clarity of emphasized content. These initial concepts were then further aggregated into 17 emotional categories, which encompassed feelings such as happiness, a sense of achievement, interest, enjoyment, admiration, a sense of control, pride, anxiety, anger, and hopelessness (see Table 4). This structured approach facilitated a comprehensive understanding of the emotional landscape experienced by students in the mathematics learning context. Phase 2 involves axial coding, also referred to as relational coding. This process involves establishing organic connections between concepts and categories in order to distill higher-level categories. In this study, the 17 sub-categories obtained from open coding were systematically organized and categorized according to the Control-Value Theory of mathematical academic emotions, ultimately resulting in the identification of six main categories: enjoyment, pride, shame, anger, and hopelessness (see Table 2). Phase 3 is identified as selective coding, also known as core coding. This phase builds upon the foundations established during open coding and axial coding, focusing on extracting and synthesizing insights based on the inherent logic among the primary emotional categories. Through iterative exploration and analysis of both the primary and secondary categories, this study ultimately classifies the core category as “Emotional Types and Emotional Response Mechanisms of Students in Mathematics Learning.” This classification provides a comprehensive understanding of the emotional landscape that students experience during their mathematics learning journey.

**Table 4:** Results of open coding.

Emotional concept categories	Initial concepts	Frequencies
Happy	Solving problems; teacher praise; simple tasks; peer discussions; engaging in interesting content	15
Sense of achievement	Successful self-study; instructing classmates; completing homework correctly; surpassing personal limits	8
Interest	Humorous teaching; enthusiasm during classes; teachers’ clear instruction; abundance of teaching ideas	12
Enjoyment	Active classroom atmosphere; friendly classmates; encountering exam questions taught before; good classroom discipline	4
Admiration	Logical reasoning; difficulty deconstruction; clear problem-solving approaches; neat board work; drawing circles freehand; serious attitude; employing multiple methods to explain problems	11
Sense of control	Ability to learn independently; timely and accurate homework completion; good examination results; serving as a peer tutor	8
Pride	Better performance than classmates; unexpected surprises; outstanding performance; teacher affirmation	5
Boredom	Feeling drowsy during class; unable to understand mathematics contents; frustration with mathematics; procrastination on assignments; absent-mindedness	6
Embarrassment	Being ridiculed by the teacher; being given a nickname; public criticism; being wrongfully corrected; excessive confidence	10
Anger	Frequent calculation errors; blame from parents; poor performance in mathematics; punitive measures such as standing or corporal punishment	12
Anxiety	Mathematics examinations; ranking; answer questions in front of the classmates; announce mathematics results to public	11
Fear	Punishment from parents; peer disrespect; declining performance in mathematics; mass distribution of grades by teacher	8
Despair	Inability to improve; perceived lack of academic aptitude; potential difficulty in gaining university admission	6
Helplessness	Neglect by the teacher; willful blindness; perceived hopelessness	4
Sadness	Criticism for poor performance; cold attitude from the teacher; unfair treatment; preferential treatment towards others	4
Rage	Arbitrary punishments; favoritism; simultaneous punishment and parent notification; overly strict management; emotional biases in the classroom; targeting specific students	9
Contempt	Teacher’s inability to solve problems; incorrect instruction; venting frustration on students; let students self-directed study during class; teacher’s distraction with personal devices	2
Indifference	Perceived inability to learn; lack of expectations; teacher’s inability to manage; a mindset of just getting by.	4

#### 3.2.2 Acoustic analysis: parameters, model validation and reliability

Audio data were sourced from approximately 32 videotaped lessons (totaling approximately 1,280 min) delivered by Chinese mathematics teachers exhibiting dominant interpersonal behavior. All recordings were conducted in naturalistic classroom settings during regular instruction, rather than under staged or experimental conditions. Selection of audio segments for analysis followed three purposive criteria. First, segments were included only if they contained explicit verbal interactions between the target teachers and student participants. Second, the selected segments (duration greater than 3 s and less than 90 s) had to exemplify the teacher’s dominant interpersonal style, as operationalized in Section 2.1. Third, and critically, the student speakers in each selected segment had to be among the 16 participants who completed the CIT interviews. A total of 48 teacher–student interaction episodes met the inclusion criteria, with an average duration of 35 s per episode (total analyzed interaction time ≈ 28 min).

Multimodal voice data were analyzed using openSMILE software (version 3.0.2). In order to enable objective classification of student emotions from classroom speech, we first established reference intervals for six prototypical emotions using the CASIA Chinese emotional speech database (Institute of Automation, Chinese Academy of Sciences). This database contains approximately 1,200 utterances from four speakers, spanning six emotion categories: neutral, happy, sad, angry, fearful, and surprised. For each utterance in CASIA, we extracted three core acoustic features using openSMILE 3.0.2: (1) Mean pitch (F0semitoneFrom27.5Hz_sma3nz_amean); (2) Mean loudness (auditory loudness); (3) Spectral flux (spectralFlux_sma3nz). These features were aggregated per emotion category across multiple speakers. Table 1 reports the 5th–95th percentile ranges for each emotion, serving as the reference matrix for subsequent classroom emotion recognition. These intervals are consistent with prior acoustic emotion recognition literature (D’Mello and Gruber, 2021; Wu et al., 2025) and provide a transparent, reproducible decision boundary.

Subsequently, audio extracts from the classroom interaction recordings of the 48 selected teacher–student episodes were processed using the same openSMILE protocol to extract identical LLDs, including pitch, speech rate, energy, and spectral tilt, with corresponding functional aggregations. The extracted acoustic features from the classroom data were then compared against the CASIA-derived reference matrix to assign emotion classifications (e.g., happy, sad, angry, fearful) to each speech segment. To align with the theoretical framework of this study, the CASIA emotion categories were mapped onto the five achievement emotions identified in the CIT analysis: “happy” was mapped to enjoyment and pride; “sad” and “fearful” were mapped to shame and hopelessness; and “angry” was mapped to anger. This mapping is theoretically grounded in control-value theory’s two-dimensional structure of valence (positive/negative) and arousal (high/low activation) (Pekrun, 2006). For positive emotions, both enjoyment and pride are high-arousal positive states. Following Ekman (1992) basic emotion framework, “happy” serves as the prototypical positive emotion. Empirical studies have confirmed that expressions of happiness, joy, and pride share convergent acoustic features, including higher loudness, greater pitch variability, and increased energy (Kamiloğlu et al., 2020; Cavanaugh et al., 2016). Moreover, the recognizability of vocal positive emotions (i.e., happiness, joy, and pride) have been validated across different cultural and linguistic populations, supporting the cross-context generalizability of this acoustic signature (Anikin and Persson, 2016; Laukka et al., 2016). For negative emotions, control-value theory distinguishes between high-arousal states (e.g., shame, anxiety) and low-arousal states (e.g., hopelessness, sadness). We therefore map CASIA “fearful” (high-arousal negative) to shame, and CASIA “sad” (low-arousal negative) to hopelessness (Scherer, 2003; Bänziger et al., 2012). This mapping is an analytical approximation rather than a direct equivalence, and its validity is strengthened through triangulation with CIT interview data (see Table 2).

The CASIA database and openSMILE feature extraction approach have been validated in prior research on spontaneous speech, demonstrating good convergent validity with human coders (D’Mello and Gruber, 2021; Wu et al., 2025). To ensure contextual validity, a random sample of 20% of the annotated segments was independently coded by two trained research assistants, yielding an inter-rater agreement of *κ* = 0.81 for emotion categories, indicating strong reliability. Finally, multimodal data fusion was conducted through comprehensive analysis and triangulation with CIT-derived data.

All data in this study were transcribed by the author and then analyzed through a qualitative inductive process. Three main steps were conducted in data analysis. First, to gain insight into each participant’s overall impression, the author meticulously examined the complete transcripts and formed initial impressions regarding the students’ emotional responses. Second, thematic analysis was used to code each transcript with the assistance of Nvivo and openSMILE. With exploring the potential connections between codes and themes, several main emotion patterns emerged from students’ interview data in relation to the interaction of math teachers. Third, the author reread the transcripts carefully to identify whether some detailed and complex data was lost, until all emerging themes which reflected students’ achievement emotions in the learning processes was coded and sorted appropriately. For example, a number of in-depth emotions in this study related to students’ mathematics achievement, such as “enjoyment of mathematics learning,” “shame experienced after feedback of mathematics achievement” were derived from the participants’ indirect description, which were wealthy to further examine in accordance with the theoretical framework of “control-value” theory of achievement emotion. All qualitative data of this study were analyzed and coded by the first author, then discussed together in the research group to validate the interpretations and ensure reliability.

## 4 Results

**Figure 4:**
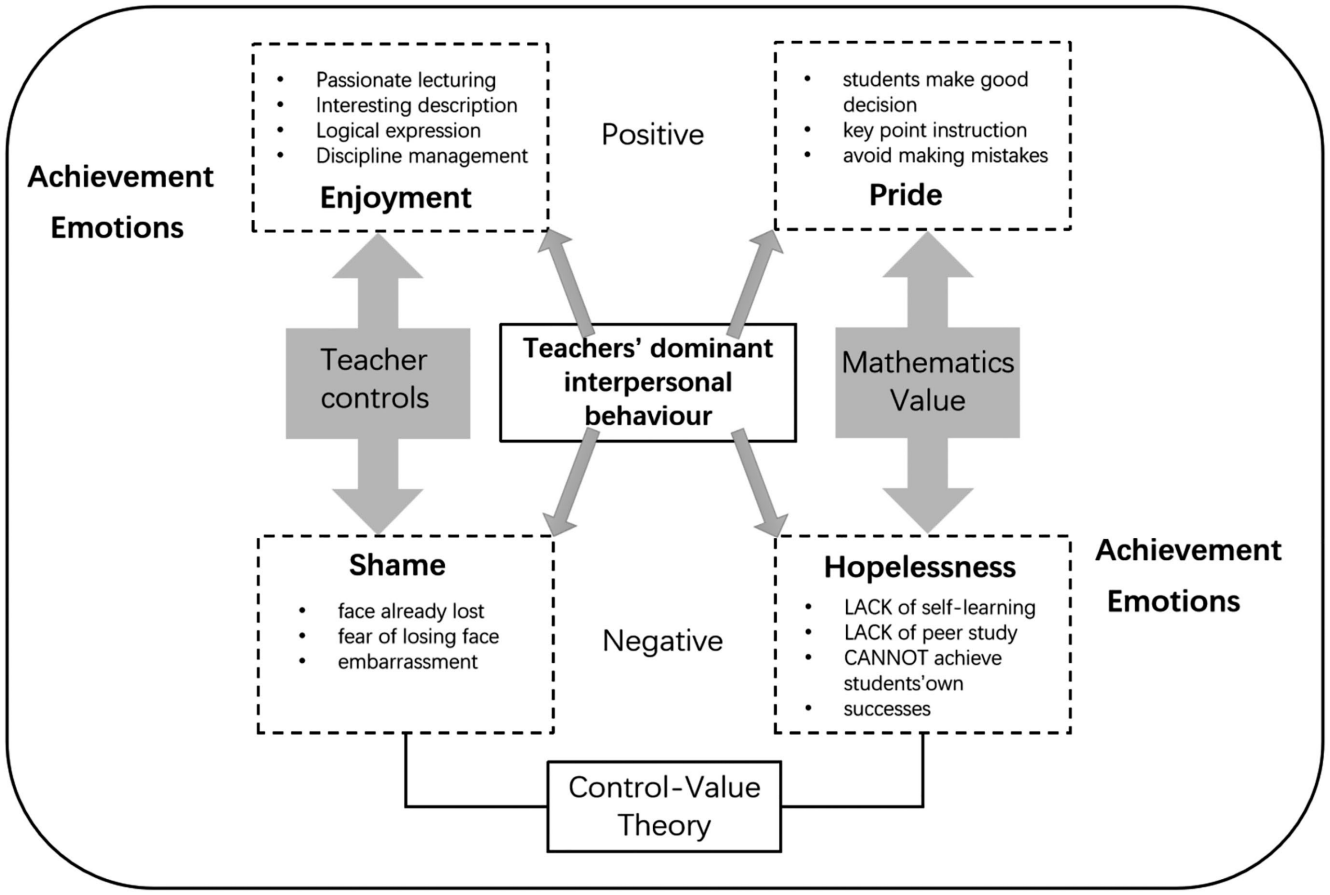
Students’ mathematics achievement emotions to teachers’ dominant interpersonal behavior.

In exploring the impact of teachers’ dominant interpersonal behaviors teaching on students’ mathematics achievement emotions, emphasis was placed on examining the emotional responses of students within mathematics classrooms. Figure 4 summarizes students’ various emotions to teachers’ dominant interpersonal behavior and the specific reasons.

### 4.1 Enjoyment: “teacher ‘controls’ our whole class mathematics learning”

The study found that some of the participants obtained enjoyment in mathematics classes from teachers’ whole class controlling through passionate lecturing and interesting description. They portrayed amusing anecdotes where teachers demonstrated their wit through unique metaphors, a blend of Chinese and humorous English, and even employed comedic dialogue to elucidate certain mathematical problems. (Jack Round 1).

I enjoy my math teacher’s classes very much because he is energetic and engaging. He explains mathematical concepts in a way that is easy to understand, which helps me learn quickly. But if one of us steps away, the other two cannot maintain that shape.” Through this interactive demonstration, he helped us vividly understand the concept of the triangle’s stability. (Ben Round 1).

In addition, some students enjoyed teachers’ logical expression. For example, they were benefit from the ways “teacher guide,” which lead them “straightforward to solve the math problem,” they appreciated that “teacher gave a clear lecture so that we would not be distracted”. (David Round 1).

Other students gained enjoyments from teachers’ attitude instruction and discipline management. They impressed that “our teacher not only teaches us math problems in class, but also cultivates our attitudes to life.” These optimistic perspectives and attitude towards learning and life encouraged them to learn. Students also impressed by “teacher’s sharp eyes,” which make sure “all students to concentrate on teaches in mathematics classes, no one bow to do their own things”. (Ben Round 2).

Our math class is very active because the teacher has a great sense of humor. If someone is not paying attention, the teacher will either give them a stern look or use a humorous approach to regain their attention. I think his way of managing discipline is very effective. (Ben Round 2).

### 4.2 Pride: “I believe that my teacher can help me learn mathematics well”

On the other hand, some students experienced a sense of pride upon receiving feedback on their mathematics learning, which was facilitated by the guidance and leadership of their teachers. For instance, teacher’s key point instruction was rather impressive. Students conveyed that “it’s good for teachers to lead us thinking in the key place. Training in this way, we will be sensitive to these key positions in problem solving processes” (Jack Round 2). Under the guidance of teachers’ instructions, students’ problem-solving abilities can be enhanced progressively.

When we come to difficult problems, teacher will point out some key points to us. Similarly, sometimes we come across difficult geometry problems, teacher will point out an important “auxiliary line” to us. At the same time, he will analyze how to figure out the auxiliary line by the known conditions. Training to find the key points of math problems for a long time, we will be sensitive to these key positions. We are very happy to see the familiar key points in the exams! (Jack Round 2).

Moreover, they take pride in avoiding mistakes after following teacher’ instructions.

Students explained that “teacher will help us to avoid the detour in time. Wherever we met a roundabout way, teachers will point out to us from this part, which give us a really deep impression”. (David Round 2).

I appreciate that our math teacher takes the time to review each of our mistakes after class. Whenever we make errors in our homework, he asks us to stay in the classroom so he can help us address them promptly. (Susan Round 1).

### 4.3 Shame: “I’m scared of teacher’s judgement about me”

Shame is an emotion generated in social relationship. By teachers’ transmission instruction, students sometimes felt ashamed when they perceived that their teacher was making a negative judgment about them (Wang and Li, 2003). Three main categories of shame experiences were found in students’ mathematics learning processes. Firstly, face already lost. This emotion associated with reactions after faces lost. For example, students expressed dissatisfaction with negative interactions from teachers following their failure in mathematics exams. The use of punishment or sarcasm by teachers left them feeling depressed and reluctant to participate in future discussions. (Susan Round 1).

I struggle with mathematics, and my classmates often laugh at me because of my low grades. This is especially true when the teacher announces our scores in class and some students laugh out loud. The more they laugh at me, the less motivated I feel to study mathematics. (Hugo Round 1).

Secondly, fear of losing face, which is concentrated on the psychological and physiological reactions before faces lost. Students were particularly afraid about teachers reading out their math results in class or sending the grades to the parents, especially when they failed in the exam. (Jane Round 1).

All of my classmates do not want teacher to send math grades to parents. I remember once I did not do well in the exam, teacher sent the whole class score to parents. At that time, I was very angry that the teacher sent all the grades to my parents group, which will make my parents pay more attention to the grades of other students rather than my mistakes. (Jane Round 1).

Thirdly, embarrassment. This emotion tied to the students’ reactions by teachers attempting to embarrass them.

I often find it difficult to understand anything in math class, so I tend to keep to myself during lessons. Sometimes the teacher ignores me; at other times, I am punished by being made to stand in front of the class. The more embarrassed I become, the harder it is for me to understand mathematics. (Hugo Round 2).

When students were punished by their teachers, most of them felt very humble. They were unable to follow what teacher said later, which impeded their mathematics learning seriously (Ricky Round 1). Especially, students felt mathematics learning as a hopeless task when they suffered double punishment from both teachers and parents. (Susan Round 2).

I still remember the time I failed a math test in sixth grade. The teacher was so angry that she called my parents to the school and spoke with them for a long time. I felt that the teacher had not taught well, and even worse, I was being punished twice—both at school and at home. This made me feel hopeless about ever being able to learn math well. (Susan Round 2).

### 4.4 Hopelessness: “I nearly cannot realize the value of learning mathematics”

Some participants expressed that they struggle to find excitement or hope in high-control teacher interaction behaviors during mathematics classes due to the lack of opportunities for self-directed learning. This absence makes it difficult for them to recognize the value of mathematics education. Participants suggested that successful self-learning experiences could enhance students’ control over their mathematics learning. Additionally, students in this study noted that teachers who employ high-control interaction methods often do not allow sufficient time for them to think through problems before providing explanations. As a result, students frequently find it challenging to articulate their own questions, which hinders their ability to fully understand the teacher’s instructions. (Gloria, Round 1).

Moreover, peer study included peer discussion, small teacher role play, and peer double check which would help students gain enjoyment were seldom utilized in high-control teacher interaction teaching. Seeking assistance from peers was particularly beneficial for students when they faced challenging problems (Jane, Round 1). In contrast to the constant lecturing by teachers, when students take on a teaching role or assist their peers, it often leaves a positive impression. (Tony, Round 1).

I also enjoy discussion time with my classmates, because it allows us to share different perspectives and sometimes a classmate can help me find a breakthrough to solve a problem. However, when two classmates are discussing or checking answers together, it is harder to get distracted. Moreover, having discussions encourages us to think ahead and prepare our ideas, which helps us understand the teacher’s lecture better. (Gloria Round 2).

Students also complained that high-control interaction teaching impedes their ability to achieve personal success in solving mathematics problems and diminishes their experiences of independently outperforming their classmates. Many students felt proud of their accomplishments in mathematics when they understood the learning content (Tony, Round 2) or successfully solved problems they previously found challenging (Ricky, Round 2). One student articulated, “If I solve a difficult problem while other students have not done it yet, I feel really excited. If the teacher had not given us the chance to discuss it, I might not have had that feeling”. (Angela, Round 1).

I do not like that our math teacher never gives us a chance to try solving problems on our own first. However, our teacher leads us through everything in class, so we have become too dependent on her. When I try to solve problems by myself, I feel a sense of achievement. On the other hand, when the teacher just keeps talking, I feel bored. (Gloria Round 2).

Instead of instilling a sense of “hope for success,” many participants noted that traditional transmission-based instruction made them feel a sense of “hopelessness due to failures. “Students discovered that when given opportunities to make decisions, they felt more confident in achieving success. In contrast, teachers’ dominant interpersonal behaviors resulted in students relying solely on teachers’ decision-making, neglecting the consideration of other students’ ideas, which left them feeling dissatisfied.

I hope our math teacher can try sort out the problems by following my original thinking, and continue to think according to my direction. I’m very happy to think that I could solve the problem in my own way. I think this is not only teacher give me space to solve the problem, but also give me the chance to understand why I need to learn math. (David Round 2).

### 4.5 Multimodal voice analysis results: acoustic correlates of dominant interactions

To complement the thematic findings from the CIT analysis, this section presents the results of the objective acoustic analysis conducted using openSMILE. For each student speech segment extracted from classroom recordings, we computed the same three acoustic features (mean pitch, mean loudness, spectral flux). A segment was assigned to an emotion category if all three feature values fell inside the corresponding CASIA-derived reference interval (p05–p95). Segments with features crossing multiple interval boundaries were excluded from categorical analysis (<6% of all segments). The findings are structured to illustrate how specific vocal patterns in teacher-student interactions correspond to the achievement emotions identified qualitatively.

#### 4.5.1 Acoustic profile of a teacher’s “dominant” tone

Aggregate acoustic analysis of teacher speech across the recorded incidents revealed two distinct profiles of dominant interpersonal behavior, which we term Cooperative (Supportive) Dominance and Opposite (Authoritarian) Dominance. Profiles were derived from aggregate acoustic features that showed statistically significant differences (*p* < 0.05) between the two dominance types (see Table 5).

**Table 5:** Acoustic features of teachers’ dominant interpersonal behavior profiles.

Acoustic feature	Cooperative (supportive) dominance profile	Opposite (authoritarian) dominance profile
Mean pitch (F0)	Moderate, within a comfortable range.	Significantly higher (often > + 1.5 SD from baseline).
Pitch variability	High, using melodic intonation for emphasis.	Low to moderate, with a flatter, more monotonous contour.
Speech rate	Moderate and steady.	Fast and often rushed.
Spectral energy (high-freq.)	Balanced, indicating a clear, projected voice.	Often heightened, indicating vocal tension or strain.
Inferred paralinguistic tone	Guiding, encouraging, clarifying.	Impatient, pressuring, interrogative.

#### 4.5.2 Triangulation of vocal and qualitative data: case illustrations

The following cases demonstrate how the acoustic data triangulates with and enriches the qualitative interview findings.

Case A: Triangulating “Pride” (Student: Ben).

CIT interview excerpt: “I was very happy to share my solution. I felt proud that the teacher chose me.”

Interaction context: Teacher invites Ben to explain his problem-solving approach to the class.

Vocal analysis results:

Teacher’s speech: Exhibited a Supportive Dominance profile. Mean pitch increased moderately to convey interest, and speech was fluent and clear.

Ben’s speech: Mean pitch(272 Hz) falls inside pride interval (150–361 Hz); Mean loudness(0.68) falls inside pride (0.301–1.077); Spectral flux(0.25) falls inside pride (0.128–0.928).

Arousal: High (evidenced by elevated but controlled pitch variability and speech rate).

Valence: Positive (indicated by a balanced spectral centroid and high, stable energy).

Inferred emotional state: “Excited,” “Confident.”

Interpretive synthesis: The objective vocal data—showing high activation and positive valence—provides convergent evidence for Ben’s self-reported feelings of pride and enjoyment. The teacher’s supportive vocal framing facilitated a positive emotional expression.

Case B: Triangulating “Shame” (Student: Hugo).

CIT interview excerpt: “His voice was sharp. I just wanted to be quiet so he’d move on. I felt so embarrassed.”

Interaction context: Teacher rapidly fires a series of questions at Hugo after he fails to answer the first one.

Vocal analysis results:

Teacher’s speech: Exhibited an Authoritarian Dominance profile. Mean pitch was high and sharp, speech rate was fast, and spectral features indicated tension.

Hugo’s speech: Mean pitch (258 Hz) falls inside shame interval (114–312 Hz), but also overlaps with anger, while loudness and flux indicate low control; mean loudness (0.31) only inside shame (0.315–0.806); spectral flux(0.14) falls inside shame (0.122–0.493).

Arousal: High (evidenced by a high density of pauses and disfluencies, a sign of cognitive stress).

Valence: Strongly negative (indicated by very low acoustic energy and a drop in mean pitch).

Inferred emotional state: “Anxious,” “Withdrawn.”

Interpretive synthesis: Hugo’s vocal signature of high negative arousal aligns perfectly with his account of experiencing shame and anxiety. The teacher’s authoritarian vocal tone preceded and likely triggered this negative vocal-emotional response.

The integration of open SMILE-based acoustic analysis with CIT findings provides robust, objective evidence that teachers’ dominant interpersonal behaviors are not monolithic. The specific acoustic delivery of dominance—whether supportive or authoritarian—is a critical determinant in eliciting fundamentally different achievement emotions in students, thereby validating and refining the emotional paradox identified in this study. The results reveal ambivalent emotional responses to teachers’ dominant interpersonal behavior, modulated by paralanguage, specifically, the distinction between supportive and authoritarian vocal tones. These findings are discussed in relation to control-value theory, and their implications for mathematics teaching are addressed.

## 5 Discussion

### 5.1 Self-contradiction and ambivalent emotions: students’ emotional responses to teachers’ dominant interpersonal behaviors

This study found that students’ emotional responses to teachers’ dominant interpersonal behavior were interpreted by participants as being shaped by their interactions with math teachers. Positively, students felt enjoyment and pride when such methods helped them grasp mathematical concepts. However, these methods often led to feelings of hopelessness and shame when they negatively affected students’ self-assessment of their performance. According to control-value theory, students’ achievement emotions in mathematics are influenced by messages about success and failure from key figures like teachers. Their prospective emotions (e.g., how they feel about possible outcomes) are closely linked to their perceptions of success, failure, and the consequences of these outcomes (Pekrun et al., 2010). The ambivalent emotional patterns summarized in Figure 2 can be explicitly linked to the acoustic parameters of teachers’ dominant interpersonal behavior presented in Table 5. Specifically, the cooperative (supportive) tone identified in Figure 2, operationalized in Table 5 as moderate pitch, stable energy, and fluent pacing, is hypothesized to enhance students’ perceived control and positive value appraisals. According to control-value theory (Pekrun, 2006), this combination of high control and positive value is associated with activity-related enjoyment and outcome-related pride. In contrast, the oppositional (authoritarian) tone in Figure 2, operationalized in Table 5 as high/sharp pitch, vocal tension, and rushed speech, is hypothesized to undermine perceived control and elicit negative value appraisals, thereby being associated with outcome-related shame and hopelessness. Thus, the same dominant interpersonal behavior (teacher control) may produce divergent emotional outcomes depending on its acoustic delivery, which shapes students’ control and value appraisals.

In this study, assessing teachers’ dominant interpersonal behavior’s impact requires examining students’ prospective emotions toward external controls. For instance, “anticipatory joy” occurs when teachers firmly control learning materials and set high expectations for success (Westphal et al., 2018). In contrast, “hopeless anxiety” arises when students focus on possible failures in high-stakes math exams, increasing their anxiety (Chen and Brown, 2018). This moderate anxiety can intensify if students do not use strategies to build hope.

Chinese students often face intense math anxiety and pressure while aiming for high grades in end-of-school entrance exams (Sun, 2011). Research shows that students’ enjoyment is heavily shaped by external factors like classroom management, teaching methods, and support (Kunter et al., 2013). On the other hand, anger is more directly triggered by external actions, such as unclear instructions from teachers (Harley et al., 2019). Chinese learners are deeply influenced by high power distance and Confucian philosophy, which establish a formal hierarchical relationship between teachers and students (Alqarni, 2022). They view teachers as authoritative sources of knowledge and believe that the teacher-student relationship greatly impacts their motivation and learning outcomes (Tran et al., 2016). This often results in strong academic and psychological dependence on teachers. Additionally, shaped by collectivist values, Chinese learners see individuals as inseparable from the group. Their adherence to group norms is often driven not by fear of authority but by a desire to integrate smoothly into the group (Tran et al., 2016). This may be because students prioritize shielding themselves from external challenges rather than addressing internal issues (Raccanello et al., 2018). This ambivalence can be resolved by differentiating teachers’ instructional behaviors (instructional structure) from their relational styles (interpersonal dominance). This conceptual distinction also offers practical implications for daily mathematics teaching.

### 5.2 Teacher control and value judgments: the critical role of dominant interpersonal behaviors in shaping students’ emotions

Students’ emotions in mathematics learning are influenced not only by their environment but also by their value judgments regarding their learning processes (Pekrun et al., 2010; Pekrun and Perry, 2014). In this study, students’ prospective emotions about outcomes align with teachers’ dominant interpersonal behaviors, specifically variation teaching. For example, students noted that teachers helped them distinguish key learning points from related questions, grasp the essence of problem-solving, and sharpen their sensitivity to question types. Successfully solving such problems during exams brought them latent joy and anticipatory pleasure, boosting their confidence in math study. Conversely, students expressed anger, saying: “I get angry when I fail to finish homework or encounter difficult problems, especially when scolded by my math teacher” (Daisy). This highlights how external factors, including the environment and teacher behavior, can trigger negative emotions. Therefore, the emotional effects of prospective outcomes should be accounted for, alongside other controls and interventions, within dominant interpersonal behaviors methods depending on the specific event.

“Control-independent emotions” refer to achievement emotions shaped by students’ internal self-control values, such as self-efficacy and academic self-concept. Research shows that emotions like pride and shame in mathematics are strongly linked to these individual control values (Jansen et al., 2015). For example, when students struggle with math problems, they often blame internal factors beyond their control, such as a lack of connection to the subject’s value or insufficient personal effort. This internal conflict frequently results in feelings of shame. Further, students found it hard to form their own opinions about what success and failure meant. Because of this, they lacked independence and struggled to solve challenging math problems on their own, which led to negative feelings when looking back on their learning experiences. Their emotions were also affected by teachers making all decisions independently, limiting students’ involvement in the learning process. The study also found that students often experienced “attribution-independent emotions,” which are directly linked to their perceptions of past successes or failures and typically arise after math-related learning events. Research suggests that feelings of competence play a key role in shaping students’ emotions (Raccanello et al., 2018). In the context of math learning, students’ perceived value refers to how much they personally believe math is relevant or meaningful to their goals (Pekrun and Perry, 2014). The negative emotions observed among students in this study stemmed from two main factors: a lack of successful learning experiences and their inability to recognize the importance or value of math in their personal growth and future aspirations.

Under the cultural influence of Chinese learners, math students often exhibit a complex dynamic of relying on teachers while striving for independence, driven by three key factors. First, the authoritative role of teachers can create feelings of shyness or fear among students, causing some to avoid close interaction to escape criticism or unwanted attention (Tran et al., 2016). Second, in collectivist societies, social standing and group recognition are highly valued. Students, eager to gain peer approval and preserve their sense of dignity, may distance themselves from teachers to display independence and capability, especially during group collaboration (Alqarni, 2022). Finally, faced with increasingly competitive academic environments, students are developing stronger independent thinking skills, relying less on teachers and prioritizing self-driven exploration as a means to gain a competitive edge (Tan et al., 2021). These factors highlight the interplay between cultural values, teacher-student dynamics, and evolving learner independence.

### 5.3 Limitations

Several limitations of the present study should be acknowledged. First, the sample comprised 16 secondary students from four schools in Shenzhen, China. While purposive sampling ensured representation across diverse school types (private, public, prestigious, and nine-year compulsory education schools), the relatively small sample size and restriction to a single metropolitan region limit the generalizability of the findings to other populations, including rural students or those from different provincial educational systems in China.

Second, the study employed a qualitative, non-causal design. The Critical Incident Technique (CIT) and multimodal voice analysis, while offering rich, contextualized insights into students’ emotional experiences, do not permit causal inferences regarding the direction of effects between teacher vocal tone and student emotions. CIT relies on retrospective accounts, which may introduce recall bias and social desirability bias (Flanagan, 1954). Furthermore, acoustic signals are indirect proxies for emotional states, and the mapping from basic emotions to achievement emotions is an analytical approximation rather than a direct equivalence. As such, emotion recognition from voice is probabilistic rather than definitive, with a reported model accuracy of 72.3% (D’Mello and Gruber, 2021), and acoustic interpretation is inevitably shaped by contextual assumptions, although triangulation with interview data partially mitigates this concern. It also remains possible that students’ pre-existing emotional states influenced their perceptions of teachers’ dominant interpersonal behavior, or that unmeasured third variables (e.g., student ability, teacher personality, classroom climate) confounded the observed associations.

Third, the cultural embeddedness of teacher authority and collectivist values in China means that the extent to which these findings transfer to other contexts remains uncertain. Transferability is most plausible to other East Asian or high-power-distance educational settings, but this requires empirical validation through future cross-cultural research. Readers are therefore cautioned against direct generalization to Western, low-power-distance classrooms.

### 5.4 Future directions

Based on these limitations, future research should pursue four directions. First, longitudinal designs are needed to examine how teachers’ dominant interpersonal behavior shapes emotions over time and to establish temporal precedence. Second, cross-cultural comparative research (e.g., China versus Western contexts) would help disentangle universal from culturally specific mechanisms. Third, larger and more diverse samples (including rural schools and different grade levels) would enable quantitative testing of the proposed mediation pathways. Fourth, experimental designs that manipulate vocal parameters (e.g., pitch and pacing) could help establish causal effects, while extension to other subjects and additional channels (e.g., facial expression and physiological measures) would further validate the multimodal framework.

## 6 Conclusion

This study set out to explore the emotional responses students exhibit towards teachers’ dominant interpersonal behaviors (RQ1) and the mechanisms through which these behaviors influence achievement emotions (RQ2). By employing a multimodal, qualitative approach, we have demonstrated that the answers are not linear (Pekrun and Bühner, 2014). Students’ emotions are complex and often contradictory, shaped profoundly by whether dominance is acoustically framed as supportive guidance or authoritarian control. This refines the control-value theory by highlighting the interpersonal style as a critical antecedent to control and value appraisals.

Focusing on early adolescents answers calls for age-sensitive research and clarifies how developmental stage shapes the perception and impact of teacher control and care (Boukari et al., 2022). By sampling students of the same region and age across different school types and attainment levels, the study situates emotional responses within culturally specific meanings of authority, thereby refining cross-cultural debates that report mixed affective outcomes under teachers’ dominant interpersonal behavior (e.g., no differences across contexts, or higher enjoyment and anxiety among Chinese students; Jia and Cheng, 2024). Conceptually and practically, the study reframes teacher-centred instruction as contingent on interpersonal style and cultural meaning showing when clarity, enthusiasm, and monitoring with care can sustain positive emotions and specifies the mechanisms and conditions under which teacher-led practices shape learners’ emotions in mathematics.

Mathematics teachers are encouraged to employ a variety of pedagogical strategies to cultivate a positive emotional climate in the classroom, while maintaining flexibility in their instructional practices. For instance, variation teaching can be integrated into teacher-domained mathematics instruction, whereby teachers introduce variations in nonessential knowledge (such as specific examples) to emphasize and clarify the essential concepts by highlighting contrasts (Gu, 2010). In order to balance teacher control with students’ sense of value, it is recommended that teachers focus on five key areas: balancing knowledge delivery with student comprehension, connecting the curriculum to real-life applications, clearly defining the roles of both teachers and students, sharing responsibility for learning, and integrating both summative and formative assessments. Additionally, teachers should actively support students in managing negative emotions by adjusting instructional strategies to accommodate individual needs. Achieving this balance is crucial for effective mathematics teaching, as it addresses the “Chinese learner paradox” and promotes more effective implementation of teachers’ dominant interpersonal behaviors.

## Data Availability

The original contributions presented in the study are included in the article/supplementary material, further inquiries can be directed to the corresponding author.

## References

[ref1] Alqarni A. M. (2022). Hofstede’s cultural dimensions in relation to learning behaviours and learning styles: a critical analysis of studies under different cultural and language learning environments. J. Lang. Linguist. Stud. 18, 721–739.

[ref2] Anikin A. Persson T. (2016). Nonlinguistic vocalizations from online amateur videos for emotion research: a validated corpus. Behav Res Methods 49, 758–771. doi: 10.3758/s13428-016-0736-y 27130172

[ref3] Baghoussi M. (2021). Teachers-centered approach interactions prevalence in Algerian secondary-school EFL classes: the case of English teachers and learners in Mostaganem district. Arab World Engl. J. 12, 268–278.

[ref4] Bänziger T. Mortillaro M. Scherer K. R. (2012). Introducing the Geneva multimodal expression corpus for experimental research on emotion perception. Emotion 12, 1161–1179. doi: 10.1037/a0025827, 22081890

[ref5] Bature I. J. Campus C. (2020). The mathematics teachers shift from the traditional teacher-centred classroom to a more constructivist student-centred epistemology. Open Access Libr. J. 7, 1–26. doi: 10.4236/OALIB.1106389

[ref7] Boukari S. Guelmami N. Chotrane S. G. Bouzid S. Khemiri A. Muscella A. . (2022). Adaptation of the questionnaire on teacher interaction in Tunisia: teaching strategies to promote sustainable education in schools. Sustainability 14:2489. doi: 10.3390/su14052489

[ref8] Cavanaugh L. A. MacInnis D. J. Weiss A. M. (2016). Perceptual dimensions differentiate emotions. Cognit. Emot. 30, 1430–1445. doi: 10.1080/02699931.2015.1070119, 26308182

[ref9] Chen J. Brown G. T. (2018). Chinese secondary school students’ conceptions of assessment and achievement emotions: endorsed purposes lead to positive and negative feelings. Asia Pac. J. Educ. 38, 91–109. doi: 10.1080/02188791.2018.1423951

[ref10] Cheng H. Y. Ding Q. T. (2021). Examining the behavioral features of Chinese teachers and students in the learner-centered instruction. Eur. J. Psychol. Educ. 36, 169–186. doi: 10.1007/s10212-020-00469-2

[ref11] Choi J. Han H. (2023). Understanding the influence of teacher-student relationship on mathematics achievement: evidence from Korean students. SAGE Open 13:21582440231208548-21582440231208548. doi: 10.1177/21582440231208548

[ref12] Cuisinier F. Clavel C. de Rosnay M. Pons F. (2010). “Emotions in research and practice” in Emotional Experiences at Elementary School: Theoretical and Pragmatic Issues. (Aalborg: Aalborg University Press). p. 175–201.

[ref13] D’Mello S. K. Gruber J. (2021). Emotional regularity: associations with personality, psychological health, and occupational outcomes. Cogn. Emot. 35, 1460–1478. doi: 10.1080/02699931.2021.1968797 34414862

[ref14] Decarli G. Zasso S. Franchin L. (2025). Could the impact of emotional states on learning in children vary with task difficulty? J. Exp. Child Psychol. 251:106122. doi: 10.1016/j.jecp.2024.106122, 39608334

[ref15] Dello-Iacovo B. (2009). Curriculum reform and ‘quality education’ in China: an overview. Int. J. Educ. Dev. 29, 241–249. doi: 10.1016/j.ijedudev.2008.02.008

[ref16] Ekman P. (1992). An argument for basic emotions. Cogn. Emot. 6, 169–200. doi: 10.1080/02699939208411068

[ref17] Emanet E. A. Kezer F. (2021). The effects of student-centered teaching methods used in mathematics courses on mathematics achievement, attitude, and anxiety: a meta-analysis study. Particip. Educ. Res. 8, 240–259. doi: 10.17275/PER.21.38.8.2

[ref18] Esakpaide G. (2026). Differential impacts of teacher-centered instruction on physics self-efficacy: a factorial analysis of gender in Nigeria. J. Plus Educ. 40, 318–343. doi: 10.24250/jpe/1/2026/GEGE/

[ref19] Flanagan J. C. (1954). The critical incident technique. Psychol. Bull. 51, 327–359. 13177800 10.1037/h0061470

[ref20] Freire P. (2018). Pedagogy of the Oppressed (50th Anniversary Edition). New York, NY: Bloomsbury Academic.

[ref3001] Ghafar Z. N. (2023). The Teacher-centered and the student-centered: a comparison of two approaches. Int. J. Arts Humanit. 1, 18–23. doi: 10.61424/ijah.v1i1.7

[ref21] Goetz T. Sticca F. Pekrun R. Murayama K. Elliot A. J. (2016). Intraindividual relations between achievement goals and discrete achievement emotions: an experience sampling approach. Learn. Instr. 41, 115–125. doi: 10.1016/j.learninstruc.2015.10.007

[ref22] Goldschmidt M. Scharfenberg F. J. Bogner F. X. (2016). Instructional efficiency of different discussion approaches in an outreach laboratory: teacher-guided versus learner-centered. J. Educ. Res. 109, 27–36. doi: 10.1080/00220671.2014.917601

[ref23] Gu Q. (2010). Variations in beliefs and practices: teaching English in cross-cultural contexts. Lang. Intercult. Commun. 10, 32–53. doi: 10.1080/14708470903377357

[ref24] Halstead J. M. Zhu C. (2009). Autonomy as an element in Chinese educational reform: a case study of English lessons in a senior high school in Beijing. Asia Pac. J. Educ. 29, 443–456. doi: 10.1080/02188790903308944

[ref25] Hao X. Y. He Y. Zhang D. X. Li X. Y. (2026). Teacher-focused approach to foster student engagement—a hierarchical linear model based on Chinese college students’ psychological capital. BMC Psychol. 14, 1–13. doi: 10.1186/s40359-025-03683-z PMC12764022 41316434

[ref26] Harley J. M. Pekrun R. Taxer J. L. Gross J. J. (2019). Emotion regulation in achievement situations: an integrated model. Educ. Psychol. 54, 106–126. doi: 10.1080/00461520.2019.1587297

[ref27] Hossain M. S. Muhammad G. (2019). Emotion recognition using deep learning approach from audio–visual emotional big data. Inf. Fusion 49, 69–78. doi: 10.1016/j.inffus.2018.09.008

[ref28] Husain D. Mariana A. (2026). “I prefer to stay silent”: students’ narratives of teacher dominance in ELT classroom. Indones. EFL J. 12, 184–198. doi: 10.25134/ieflj.v12i1.117

[ref29] Iotti N. O. Thornberg R. Longobardi C. Jungert T. (2020). “Early adolescents’ emotional and behavioral difficulties, student–teacher relationships, and motivation to defend in bullying incidents,” in Child Youth Care Forum, (New York, NY: Springer US), 59–75.

[ref30] Jansen M. Scherer R. Schroeders U. (2015). Students’ self-concept and self-efficacy in the sciences: differential relations to antecedents and educational outcomes. Contemp. Educ. Psychol. 41, 13–24. doi: 10.1016/j.cedpsych.2014.11.002

[ref31] Jia M. Cheng J. (2024). Effect of teacher social support on students’ emotions and learning engagement: a US-Chinese classroom investigation. Humanit. Soc. Sci. Commun. 11, 1–9. doi: 10.1057/s41599-024-02634-0

[ref32] Jmour N. Masmoudi S. Abdelkrim A. (2021). A new video based emotions analysis system (VEMOS): an efficient solution compared to iMotions Affectiva analysis software. Adv. Sci. Technol. Eng. Syst. J. 6, 990–1001.

[ref33] Kamiloğlu R. G. Fischer A. H. Sauter D. A. (2020). Good vibrations: a review of vocal expressions of positive emotions. Psychon. Bull. Rev. 27, 237–265. doi: 10.3758/s13423-019-01701-x, 31898261 PMC7093353

[ref34] Kunter M. Klusmann U. Baumert J. Richter D. Voss T. Hachfeld A. (2013). Professional competence of teachers: effects on instructional quality and student development. J. Educ. Psychol. 105, 805–820. doi: 10.1037/a0032583

[ref35] Laukka P. Elfenbein H. A. Thingujam N. S. Rockstuhl T. Frederick K. Chui W. . (2016). The expression and recognition of emotions in the voice across five nations. J. Pers. Soc. Psychol. 111, 686–705. doi: 10.1037/pspi0000066 27537275

[ref37] Lavy V. Schlosser A. (2011). Mechanisms and impacts of gender peer effects at school. Am. Econ. J. Appl. Econ. 3, 1–33.22199993

[ref38] Lin W. Wang W. Lai X. (2025). Should traditional teacher-centered teaching be removed from mathematics curriculum reform? Understanding mathematics achievement emotions from the perspective of Chinese students. Adv. Educ. Humanit. Soc. Sci. Res. 15, 174–174.

[ref3002] Lin W. Yin H. B. (2024). The relationship between junior secondary students’ characteristics of mathematics achievement emotions and their mathematics performance. Front. Educ. China 19, 369–384. doi: 10.3868/s110-010-024-0020-9

[ref40] Lin W. Yin H. Han J. Han J. (2020). Teacher–student interaction and Chinese students’ mathematics learning outcomes: the mediation of mathematics achievement emotions. Int. J. Environ. Res. Public Health 17, 1–17. doi: 10.3390/ijerph17134742 PMC7369935 32630336

[ref41] Loh C. Y. R. Teo T. C. (2017). Understanding Asian students learning styles, cultural influence and learning strategies. J. Educ. Soc. Policy 7, 194–210.

[ref42] Muganga L. Ssenkusu P. (2019). Teacher-centered vs. student-centered: an examination of student teachers’ perceptions about pedagogical practices at Uganda’s Makerere University. Cult. Pedagog. Inq. 11, 16–40. doi: 10.18733/cpi29481

[ref43] Peixoto F. Sanches C. Mata L. Monteiro V. (2017). “How do you feel about math?”: relationships between competence and value appraisals, achievement emotions and academic achievement. Eur. J. Psychol. Educ. 32, 385–405. doi: 10.1007/s10212-016-0299-4

[ref44] Pekrun R. (2006). The control-value theory of achievement emotions: assumptions, corollaries, and implications for educational research and practice. Educ. Psychol. Rev. 18, 315–341. doi: 10.1007/s10648-006-9029-9

[ref45] Pekrun R. Bühner M. (2014). “Self-report measures of academic emotions,” in International Handbook of Emotions in Education, (New York, NY: Taylor & Francis), 561–579.

[ref46] Pekrun R. Goetz T. Daniels L. M. Stupnisky R. H. Perry R. P. (2010). Boredom in achievement settings: exploring control-value antecedents and performance outcomes of a neglected emotion. J. Educ. Psychol. 102, 531–549. doi: 10.1037/A0019243

[ref47] Pekrun R. Perry R. P. (2014). “Control-value theory of achievement emotions,” in International Handbook of Emotions in Education, (New York, NY: Taylor & Francis), 120–141.

[ref48] Pennings H. J. Brekelmans M. Sadler P. Claessens L. C. van der Want A. C. van Tartwijk J. (2018). Interpersonal adaptation in teacher-student interaction. Learn. Instr. 55, 41–57. doi: 10.1016/j.learninstruc.2017.09.005

[ref49] Qin X. Laninga-Wijnen L. Steglich C. Zhang Y. Ren P. Veenstra R. (2025). The dominance of liking: uncovering dyadic and reputational effects of peer and perceived teacher likes and dislikes on friendship dynamics among Chinese adolescents. J. Youth Adolesc. 54, 903–916. doi: 10.1007/s10964-024-02104-5, 39477878 PMC11933165

[ref50] Raccanello D. Hallb R. Burroa R. (2018). Salience of primary and secondary school students' achievement emotions and perceived antecedents: interviews on literacy and mathematics domains. Learn. Individ. Differ. 65, 65–79. doi: 10.1016/j.lindif.2018.05.015

[ref51] Rao N. Chan C. K. (2009). “Moving beyond paradoxes: understanding Chinese learners and their teachers,” in In Revisiting the Chinese Learner: Changing Contexts, Changing Education, (Dordrecht: Springer Netherlands), 3–32.

[ref52] Reeve J. Cheon S. H. (2014). An intervention-based program of research on teachers’ motivating styles. Adv. Motiv. Achiev. 18, 297–343. doi: 10.1108/S0749-742320140000018008

[ref53] Saccardo F. Decarli G. Missagia V. I. Andrao M. Gini F. Zancanaro M. . (2024). Emotions and interactive tangible tools for math achievement in primary schools. Front. Psychol. 15:1440981. doi: 10.3389/fpsyg.2024.1440981, 39534471 PMC11554489

[ref54] Scherer K. R. (2003). Vocal communication of emotion: a review of research paradigms. Speech Comm. 40, 227–256. doi: 10.1016/S0167-6393(02)00084-5

[ref55] Scherer K. R. (2013). The nature and dynamics of relevance and valence appraisals: theoretical advances and recent evidence. Emot. Rev. 5, 150–162. doi: 10.1177/1754073912468166

[ref56] Schonert-Reichl K. A. (2017). Social and emotional learning and teachers. Futur. Child. 27, 137–155. doi: 10.1353/foc.2017.0007

[ref57] Schwerdt G. Wuppermann A. (2011). Is traditional teaching really that bad? A within-student betweensubject approach. Econ. Educ. Rev. 30, 365–379. doi: 10.1016/j.econedurev.2010.11.005

[ref58] Semeraro C. Giofrè D. Coppola G. Lucangeli D. Cassibba R. (2020). The role of cognitive and non-cognitive factors in mathematics achievement: the importance of the quality of the student-teacher relationship in middle school. PLoS One 15:e0231381. doi: 10.1371/journal.pone.0231381, 32310988 PMC7170247

[ref59] Sengupta-Irving T. Enyedy N. (2015). Why engaging in mathematical practices may explain stronger outcomes in affect and engagement: comparing student-driven with highly guided inquiry. J. Learn. Sci. 24, 550–592. doi: 10.1080/10508406.2014.928214

[ref60] Serin H. (2018). A comparison of teacher-centered and student-centered approaches in educational settings. Int. J. Soc. Sci. Educ. Stud. 5, 164–167. doi: 10.23918//ijsses.v5i1p164

[ref61] Sun F. P. (2011). *The Research of the Relationship Between Academic Emotions and Academic Achievement and Their Influencing Factors* (in Chinese). Beijing: Chinese National Knowledge Infrastructure.

[ref62] Sun X. Mainhard T. Wubbels T. (2018). Development and evaluation of a Chinese version of the questionnaire on teacher interaction (QTI). Learn. Environ. Res. 21, 1–17. doi: 10.1007/s10984-017-9243-z

[ref63] Tan T. X. Yi Z. Camras L. A. Cheng K. Li Z. Sun Y. . (2021). The effect of academic performance, individualistic and collectivistic orientation on Chinese youth’s adjustment. Soc. Psychol. Educ. 24, 1209–1229. doi: 10.1007/s11218-021-09650-x

[ref64] Tian L. Shen J. (2023). The effect of perceived teachers’ interpersonal behavior on students’ learning in physical education: a systematic review. Front. Psychol. 14:1233556. doi: 10.3389/fpsyg.2023.1233556, 37720632 PMC10499622

[ref65] Tran N. X. McDonald C. Davies M. (2016). Students’ Perspectives on Teacher-Student Relatedness in Vietnamese High Schools. Venice, Italy: Clevedon.

[ref66] Wan Z. H. Wan S. L. Zhan Y. (2022). For harmony and democracy: secondary students’ views on the value of developing critical thinking in a Confucian heritage context. Think. Skills Creat. 44, 101031–101010. doi: 10.1016/j.tsc.2022.101031, 38826717

[ref67] Wang D. (2011). The dilemma of time: student-centered teaching in the rural classroom in China. Teach. Teach. Educ. 27, 157–164. doi: 10.1016/j.tate.2010.07.012

[ref68] Wang Q. Li J. (2003). Chinese children’s self-concepts in the domains of learning and social relations. Psychol. Schs. 40, 85–101. doi: 10.1002/pits.10071

[ref69] Watkins D. A. Biggs J. B. (1996). “The Chinese learner: cultural, psychological, and contextual influences,” in Hong Kong: Comparative Education Research Centre, University of Hong Kong, (Camberwell, VIC: Australian Council for Educational Research).

[ref70] Westphal A. Kretschmann J. Gronostaj A. Vock M. (2018). More enjoyment, less anxiety and boredom: how achievement emotions relate to academic self-concept and teachers’ diagnostic skills. Learn. Individ. Differ. 62, 108–117. doi: 10.1016/j.lindif.2018.01.016

[ref71] Woods P. J. Copur-Gencturk Y. (2024). Examining the role of student-centered versus teacher-centered pedagogical approaches to self-directed learning through teaching. Teach. Teach. Educ. 138:104415. doi: 10.1016/j.tate.2023.104415

[ref72] Wu Y. Zhang S. Li P. (2025). Multi-modal emotion recognition in conversation based on prompt learning with text-audio fusion features. Sci. Rep. 15:8855. doi: 10.1038/s41598-025-89758-8, 40087340 PMC11909257

[ref73] Wubbels T. Brekelmans M. Den Brok P. Wijsman L. Mainhard T. Van Tartwijk J. (2014). “Teacher–student relationships and classroom management,” in Handbook of Classroom Management, eds. Emmer E. T. Sabornie E. J. (New York, NY: Routledge), 363–386.

[ref74] Wubbels T. Levy J. (1991). A comparison of interpersonal behavior of Dutch and American teachers. Int. J. Intercult. Relat. 15, 1–18.

[ref75] Zhang E. Y. Adamson B. (2007). “Implementing language policy: lessons from primary school English,” in Bilingual Education in China, ed. Feng A. (Multilingual Matters), 166–181.

[ref76] Zhang Q. Oetzel J. (2006). A cross-cultural test of immediacy–learning models in Chinese classrooms. Commun. Educ. 55, 313–330. doi: 10.1080/03634520600748599

[ref77] Zhou N. Lam S. F. Chan K. C. (2012). The Chinese classroom paradox: a cross-cultural comparison of teacher controlling behaviors. J. Educ. Psychol. 104:1162. doi: 10.1037/a0027609

